# The prospect of molecular therapy for Angelman syndrome and other monogenic neurologic disorders

**DOI:** 10.1186/1471-2202-15-76

**Published:** 2014-06-19

**Authors:** Barbara J Bailus, David J Segal

**Affiliations:** 1 Genome Center, MIND Institute, and Department of Biochemistry and Molecular Medicine, University of California, Davis, CA 95616, USA

**Keywords:** Artificial transcription factor, Engineered zinc finger, TALE, CRISPR, Gene regulation, Gene therapy, Blood–brain barrier, Angelman syndrome, Autism spectrum disorders

## Abstract

**Background:**

Angelman syndrome is a monogenic neurologic disorder that affects 1 in 15,000 children, and is characterized by ataxia, intellectual disability, speech impairment, sleep disorders, and seizures. The disorder is caused by loss of central nervous system expression of *UBE3A*, a gene encoding a ubiquitin ligase. Current treatments focus on the management of symptoms, as there have not been therapies to treat the underlying molecular cause of the disease. However, this outlook is evolving with advances in molecular therapies, including artificial transcription factors a class of engineered DNA-binding proteins that have the potential to target a specific site in the genome.

**Results:**

Here we review the recent progress and prospect of targeted gene expression therapies. Three main issues that must be addressed to advance toward human clinical trials are specificity, toxicity, and delivery.

**Conclusions:**

Artificial transcription factors have the potential to address these concerns on a level that meets and in some cases exceeds current small molecule therapies. We examine the possibilities of such approaches in the context of Angelman syndrome, as a template for other single-gene, neurologic disorders.

## Review

Angelman syndrome is a neurodevelopmental disorder that affects 1 in 15,000 children [1]. The disease is characterized as an autism spectrum disorder with individuals exhibiting severe mental and physical impairments, including a lack of speech and ataxia. In a normal individual, the region encoding the gene *UBE3A* is epigenetically imprinted throughout neuronal brain cells, with the maternal allele being preferentially expressed and the paternal allele silenced [2]. In Angelman syndrome, expression of the active maternal allele is lost [3]. Loss of the maternal allele, while the paternal allele remains silenced, results in a lack of *UBE3A* expression. Approximately 70% of all cases involve a large, 5–7-Mb, de-novo maternal deletion of the chromosome 15q11-q13 region, which includes the critical *UBE3A* gene [2]. The remaining known causes of Angelman syndrome involve mutations within *UBE3A* (~11%), uniparental disomy (~7%), and imprinting defects (~3%) [4]. About 10% of cases present phenotypically as Angelman syndrome but with currently unknown genetic or epigenetic causes. A gradient of severity affecting both motor function and cognitive ability is exhibited among Angelman syndrome individuals, correlating roughly with the size of the deletion. Individuals with point mutations tend to have less severe symptoms. There are no curative treatments for Angelman syndrome. Current treatments focus on behavioral and physical therapies to minimize symptoms. Drug therapies are used to control seizures and sleep disruption. However, the lack of potential therapies is rapidly changing as advances in molecular therapy that focus on altering a specific genes expression approach human clinical trials [5,6].

### Importance of Ube3a and progress made toward restoring UBE3A expression

For gene therapy to be a viable treatment option it is essential that the genetic target is known, and that there is evidence that a postnatal intervention would be beneficial. In 1997 mutations in *UBE3A* or that severely reduced expression of the maternal copy of *UBE3A* were found to be the causative for Angleman syndrome [3,7,8]. UBE3A is one of the many E3 ubiquitin ligases, which are known to add chains of ubiquitin to specific proteins and thus target them for proteasome degradation. A simple model for Angelman syndrome is that lack of UBE3A increases the concentration or persistence of its target proteins. However, more complex models would also need to consider that some E3 ligases facilitate monoubiquitination, which is associated with signaling rather than degradation, as well as evidence that UBE3A can act as a transcriptional co-regulator [9]. In 2011, a potential role for Ube3a in mouse neuronal synapse firing and long-term potentiation (LTP) was suggested by the discovery of its interactions with Arc and Ephexin 5. Arc was shown to be over-expressed in the absence of Ube3a, causing a depletion of AMPA receptors at the synapse and thus defects in synaptic plasticity, the chemical basis of learning and memory [10]. Also, the degradation of Ephexin5 was found to be mediated by Ube3a, which promoted aberrant excitatory synapse development [11]. More recently, loss of UBe3a was found to affect the cytoskeletal protein actin, providing an explanation for the known defects in dendritic spine density, LTP and learning [12]. However, there are likely many other targets and potential functions of UBE3A. Designing interventions to only one target or activity might produce only a partial benefit, and comprehensive therapy of all downstream effectors might be impractical. A more attractive therapeutic approach would be amelioration of the upstream causative event; that is, restoration of UBE3A expression.

The developmental delay of Angelman syndrome generally becomes noticeable after 6 to 12 months of age. Since the brain has been without UBE3A expression throughout development, an important consideration is whether late-stage (postnatal) intervention might have clinical value. The first study supporting that it could was based on the observation that αCaM kinase II was inhibited by phosphorylation in a mouse model of Angelman syndrome [13]. Substitutions that prevented the inhibition rescued many of the phenotypes of Angelman mice, suggesting that the major lesion was signaling and not abnormal development. Since αCaM kinase II is predominantly expressed postnatally [14,15], the study also suggested that other postnatal interventions might have efficacy.

Since the underlying genetic defects are known, gene therapy could, in principle, be applied to replace the missing components. However, replacement of the entire 5–7-Mb region that is deleted in the majority of Angelman syndrome cases is not a viable option due to size limitations imposed by most viral vectors [16]. A pragmatic approach might be to replace just the causative gene. In a mouse model of Angelman syndrome [17], hippocampal injection of recombinant adeno-associated virus serotype 9 (AAV9) carrying a cDNA of the mouse *Ube3a* produced localized restoration of Ube3a to wild-type levels [18]. The virus showed only modest distribution beyond the hippocampus, resulting in improvements in associative learning and memory defects. However, motor coordination deficits were not ameliorated by the treatment, possibly due to the AAV9 not reaching the cerebellum. Another concern of this approach is that the use of a *Ube3a* cDNA could alter the balance of the three known isoforms and result in only a partial functional restoration [19,20]. The necessity of a wide distribution and multiple isoforms of the gene is common in neurologic disorders, where a treatment with only a single localized cDNA distribution may not be sufficient for a full phenotypic rescue. The AAV study showed that a partial rescue was possible by postnatal introduction of a *Ube3a* cDNA, further supporting that the developmental changes may be reversible using late-stage interventions. This initial AAV study also encouraged the exploration of less traditional methods that could be translated to humans.

**Figure 1:**
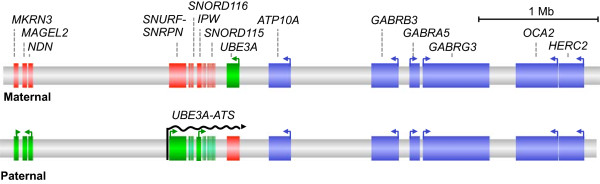
**Epigenetically imprinted genes at the Angelman locus.** The region of 15q11-13 shown is approximately that of the most common 5-Mb deletion. On the paternal allele, the green genes are expressed but the red genes are not. On the maternal allele, the imprint is reversed, green genes expressed, and red genes are not. Genes shown in blue are active on both the paternal and maternal chromosomes. Colored arrows indicate the direction of active transcription. The 600-kb brain-specific *UBE3A-ATS* transcript (indicated by black arrow) silences the paternal copy of *UBE3A*. Deletion of the maternal region therefore results in loss of *UBE3A* expression in the brain.

An attractive alternative approach for Angelman syndrome is the reactivation of the intact but silent paternal copy of *UBE3A* that exists in almost all affected individuals. Paternal *UBE3A* is epigenetically silenced not by promoter DNA methylation but by a long antisense RNA transcript referred to as the *UBE3A-ATS* (Figure 1). This 600-kb transcript initiates at the paternal *SNURF* and *SURPN* genes, extends through several clusters of small nucleolar RNAs including the *SNORD116* cluster, and continues through the paternal open reading frame of *UBE3A*[21]. This transcript is not expressed on the maternal allele, allowing expression of the maternal *UBE3A*. Although the exact mechanism of inhibition is still being elucidated, it is verified that the expression of *UBE3A-ATS* on the paternal allele severely reduces expression of the otherwise active paternal *UBE3A*[21-23].

Progress toward reactivating the paternal allele was made in a drug screen, which utilized mice that were transgenic for a Ube3a-yellow fluorescent protein fusion on the silenced paternal allele [20]. The screen showed reactivation of paternal *Ube3a* was possible through use of topoisomerase inhibitors such as topotecan [24]. Topotecan was shown to work by inhibiting transcriptional progression of the *Ube3a-ATS*, resulting in expression of paternal *Ube3a*. The drug was found to stabilize R-loop formation in the upstream *Snord116* cluster, resulting in extending chromatin decondensation but reduced transcriptional progression through the end of the *Ube3a-ATS*[24-26]. This advanced the field by confirming that reactivation of paternal *Ube3a* was possible by therapeutic targeting of the *Ube3a-ATS* transcript. The result was especially promising since topotecan was an FDA-approved anti-cancer drug. The value of re-purposing FDA-approved drugs is particularly important for rare diseases, as it is often felt that the cost of developing a new drug and bringing it successfully through full clinical trials would be financially unrealistic with the limited resources available for rare disease research. However, various complications involving specificity, toxicity, biodistribution, and delivery have impeded the advance of topoisomerase inhibitors toward human trials for Angelman syndrome treatment.

The concept that reduced *Ube3a-ATS* expression would increase paternal *Ube3a* expression was further supported by a recent study using a mouse model that contained a transcriptional termination signal inserted in the *Ube3a-ATS* gene [22]. The insertion of the poly(A) cassette in the *Ube3a-ATS* gene resulted in premature termination of *Ube3a-ATS* transcript, allowing *Ube3a* transcription to proceed without inhibition. The increased paternal *Ube3a* expression did not reach full wild type levels, but the partial increase significantly affected phenotype. The study explored the concept of the extent to which paternal *Ube3a* expression could compensate for maternal *Ube3a* loss if the compensation was present from birth. Significant improvements were observed in obesity, LTP, long-term memory, and a variety of motor skills. These findings suggest that partial expression of paternal *Ube3a* is sufficient to ameliorate several of the phenotypes associated with Angelman syndrome in mice. This mouse model was created from a single genetically modified cell, thus the same method could not be used for therapy in humans.

### Characteristics of an ideal therapy

An ideal therapy would be specific to the targeted gene (*e.g.*, *UBE3A*), exhibit low toxicity over a long period, and be deliverable by minimally invasive means. Specificity remains one of the most common and problematical issues facing potential molecular treatment. A lack of specificity is common among small molecules that work on a DNA level. For example, chromatin-modifying agents such as 5-azaC and SAHA affect DNA methylation and histone acetylation globally, rather than targeting a specific gene [27]. Topotecan was shown to affect the transcription of over 150 long genes in human neuronal cultures [25]. As the length of the size of the genes became longer than 67 kb, inhibition of transcription increased. The regulation of “off-target” genes could lead to unwanted side effects and toxicity. As with all treatment strategies, optimization of dosage and timing of treatment can be examined for possible reduction of side effects. The issue of specificity is not unique to drugs and remains a concern for “biologics” and other molecular therapies.

Another issue is the delivery of the therapeutic agent to the site of action. The delivery problem is compounded when the desired target is the entire brain and the therapeutic agent must cross the blood brain barrier or be delivered by intracranial injection [18,24,28]. Often, direct intracranial injection will only affect a small portion of the brain. Imprinting of *UBE3A* and the 15q11-q13 region has been shown to occur throughout the brain [20,29]; therefore, the limited distribution of a drug might not be sufficient for effective therapeutic outcome. Even with multiple injections and ideal spread throughout the brain, the degradation of transient therapeutic molecules would eventually require additional intracranial injections. One way to circumvent multiple injections would be to deliver a viral vector that would express the desired therapeutic agent. Viral vectors have been used for delivery of both cDNA of *Ube3a*[18] and shRNAs to topoisomerase 1 and 2 [25] in mice. AAV and integration-defective lentiviral vectors deliver non-integrating episomes that are stable and can provide nearly indefinitely expression in the non-dividing neurons [30]. However, the restricted packaging capacity of AAV limits its therapeutic application to the delivery of small cDNAs or nucleic acids. Antibodies against AAV also limit their use in humans [31]. Perhaps the largest limitation is that viral distribution is largely restricted to area around the injected region [28,32]. Viral vectors with wider distribution would be useful for fostering long-term expression throughout the brain, as would likely be the case for Angelman syndrome.

### The potential of engineered DNA-binding proteins for therapy

**Figure 2:**
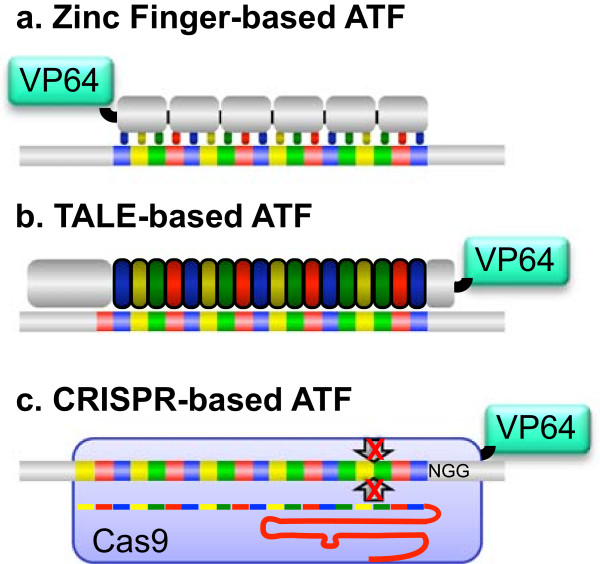
**DNA-binding platforms for artificial transcription factors. (a)** Each repeat module of an engineered zinc finger protein recognizes three base pairs of DNA. The VP64 transcriptional activation domain is shown in cyan. Other common effector domains include the KRAB repression domain. **(b)** Each repeat of a TALEN protein recognizes one base pair of DNA. A thymine base (red) just 5′ of the repeat-bound DNA site is preferred for high-affinity binding. **(c)** An engineered CRISPR system consists of a guide RNA (red strand with colored sections) that directs the Cas9 nuclease (light blue) to the target DNA. Mutation of the two-endonuclease domains (arrows with red X) produces a non-catalytic DNA-binding domain, to which an effector domain can be attached. The required protospacer adjacent motif (PAM), NGG, is shown 3′ to the target site.

For a disease caused by a single gene, the possibility of targeted gene therapy has started to gain momentum [33]. Though over 1800 gene therapy trails have been initiated since 2000, few have focused on autism spectrum disorders [34]. Angelman syndrome is one of several neurologic disorders that are caused by a single gene mutation, making it an ideal candidate for gene expression therapy. The ability to engineer DNA-binding proteins has enabled the possibility for therapeutic regulation of endogenous genes [35-38]. Such engineered DNA-binding proteins include C2H2 zinc fingers, transcription activator-like effectors (TALEs), and clustered regularly interspaced short palindromic repeat (CRISPR) systems (Figure 2). These factors can be programmed to act as activators or repressors for specific gene expression by attaching transcriptional or epigenetic effector domains, creating artificial transcription factors (ATFs) [39-42]. In the context of Angelman syndrome, such ATFs could be used to reactivate the paternal *UBE3A* that is silenced by imprinting, or inactivate the paternal *UBE3A-ATS* antisense transcript. For Prader-Willi syndrome, caused by a deletion of the paternal segment of 15q11-13, ATFs could activate the silenced maternal *UBE3A-ATS* with the goal of restoring the critical *SNORD116* transcripts that are spliced from *UBE3A-ATS*[43]. In Dup15q syndrome, caused by a segmental duplication of 15q11-13, ATFs could repress maternal *UBE3A* to reduce the doubled expression [44]. In Pitt-Hopkins syndrome, ATFs could increase expression of wild type *TCF4* in cases of *TCF4* haploinsufficiency [45]. In Rett syndrome, caused by mutation in one copy of *MECP2* on the X-chromosome, ATFs could activate the epigenetically silenced wild-type allele on the other X-chromosome in females [46].

Zinc finger and TALE-based ATFs achieve targeting specificity using amino acid side chains that recognize particular DNA base pairs through hydrogen bonding and Van der Waals interactions. A single zinc finger module is able to recognize approximately three base pairs of DNA. Considerable protein engineering efforts were able to generate modules or sets of co-evolved modules that could be assembled together to target a wide spectrum of DNA sequences [47-51]. TALEs were originally characterized from *Xanthomonas* bacteria [40,41] and contain a series of repeat modules. Each repeat uses two amino acids to recognize one base pair of DNA. The TALE repeats can be assembled in any order necessary to target a genomic site. The spectrum of sequences that can be targeted by TALEs is more versatile then that of zinc fingers. However, TALEs do not yet have the extensive animal testing and validation history of zinc fingers. No human clinical trials have been reported using TALEs, making their performance and toxicity in humans unknown.

In 2012, a new and powerful methodology called CRISPR emerged on the gene manipulation landscape [52]. This system utilizes a large protein, Cas9, which is targeted to a specific site in the genome by a guide RNA (gRNA) to create an RNA-directed double-stranded DNA nuclease. CRISPR systems are found in many archea and bacteria, where the provide immunity against invading bacteriophages and plasmids. The endonuclease activity can be inactivated by mutation and transcriptional effector domains attached to create RNA-directed ATFs [42]. This technology offers the most flexibility and expedient design of ATFs. Retargeting requires only the insertion of a new 20-nt recognition sequence in the gRNA, which can be synthesized in a time and cost efficient manner. CRISPR technology has been used successfully in numerous cell culture experiments, and the simultaneous regulation of multiple genes has been shown using Cas9 and multiple gRNAs encoded on a viral vector [38]. CRISPR technology is still in its infancy and many tests need to be conducted on the specificity and toxicity before initiating a human clinical trial. The current power of CRISPR resides in rapid synthesis of multiple ATFs and the potential to regulate multiple genes in cell or animal models.

### Challenges of utilizing DNA-binding proteins as therapeutics

One of the largest concerns for ATF-based therapy is off-target effects. It should be noted that concerns about off-target activities of ATFs are somewhat less than for nucleases; ATFs would likely have effects in only the 10-20% of the genome that contains regulatory elements [53], whereas nucleases have the potential to create mutations and rearrangements in 100% of the genome. Off-target effects have been shown to occur at variable rates for different targeting platforms. The off-target binding sites can be predicted to a large extent from *in vitro* and *in vivo* assays, making it possible to screen ATF candidates and select the most specific ones before human trials. For example, Bind-n-Seq and SELEX are methods to evaluate the most favored *in vitro* binding sites of an ATF from a random pool of all possible DNA targets [54]. Off-target sites can be predicted *in silico* based on similarity to the on-target site, then interrogated directly by analysis of epigenetic modification (if an appropriate epigenetic modification domain was used) [55] or chromatin immunoprecipitation [56]. Perhaps, the most robust *in vivo* methodology for evaluating off target binding is chromatin immunoprecipitation analyzed by high throughput sequencing (ChIP-Seq) [57]. Surprisingly, only a few studies to date have examined off-target effects by changes in non-target RNA and protein expression e.g., [58]. An ideal in-depth analysis of off-target events would involve genome wide screening for off-target binding sites by ChIP-Seq, coupled with analysis of potential off-target gene regulation by RNA-Seq and proteomics. The potential for specific on-target regulation and direct measurement of off-target effects allows for optimization of prospective therapeutic ATFs, making this approach an attractive long-term gene regulation option for clinical use.

Another important concern for all interventions is toxicity of the treatment. To date, the only ATF that has been introduced into humans is a zinc finger-based factor that increased VEGF expression, which progressed through Phase I and II clinical trials [6]. A total of 280 juvenile patients received the therapy and reported minimal adverse side effects. The ATF was delivered as a DNA plasmid injected intramuscularly. Unfortunately, little improvement was observed in patients. Efficacy aside, the clinical trials demonstrated that zinc finger ATFs were well tolerated by patients over an extended time. This tolerance, particularly the apparent lack of immune response, may have been expected since zinc fingers comprise one of the largest protein families in humans [59]. In contrast, there have been no reports yet examining the immune response of bacteria-originating TALEs or Cas9 in humans.

A significant obstacle to be overcome for every potential therapy is delivery of the therapeutic to the desired organ. As discussed above, the brain presents a unique challenge due to the blood brain barrier. Methods capable of crossing the blood brain barrier include liposomal delivery, cell penetrating peptides (CPPs), and small molecule drugs that are hydrophobic or capable of binding transcytosis proteins [60]. ATFs have been delivered to organisms through viral injections, purified protein, CPP-attached protein, and as naked coding DNA [35,36]. A potential advantage that ATFs have when delivered by a viral vector is that a protein can be coded to have a secretion and re-uptake domain, dramatically increasing its potential spread through the brain, overcoming the obstacle that cDNA vectors have of only localized viral spread [24,30]. Extensive studies have not been conducted to evaluate the potential of brain delivery of ATFs, but it is possible that one or several of these methods could prove successful.

## Conclusions

The advances that have taken place in Angelman syndrome research in the last twenty years have made it an ideal candidate for targeted molecular therapy. Recent advances have verified an intact but silenced paternal *UBE3A*. The paternal allele has been re-activated in mice by small molecule drugs that inhibit the transcription of *Ube3a-ATS*, however these drugs had several off-target effects and delivery limitations. In principle, ATFs may offer several advantages for specificity and delivery. ATFs have advanced in recent years, enabling regulation of specific gene transcripts in the human genome [58]. The delivery mechanisms of ATFs are still being optimized, but several options hold promise for crossing the blood brain barrier and long-term, gene-specific regulation. These advances may result in an eventual therapy for Angelman syndrome, allowing for the expression of paternal *UBE3A* and perhaps a full phenotypic rescue. A similar approach could be applied to other monogenetic neurologic disorders, and potentially even multi-gene disorders.

## Abbreviations

AAV: Adeno-associated virus; ATF: Artificial transcription factor; ChIP-Seq: Chromatin immunoprecipitation analyzed by high throughput sequencing; CPP: Cell penetrating peptides; CRISPR: Clustered regularly interspaced short palindromic repeat; LTP: Long-term potentiation; TALE: Transcription activator-like effector.

## Competing interests

The authors declare that they have no competing interests.

## Authors’ contributions

BJB and DJS conceived, drafted, read, and approved the manuscript.
